# Non-SARS, non-MERS human coronavirus infections and risk of Kawasaki disease: a meta-analysis

**DOI:** 10.2217/fvl-2021-0176

**Published:** 2021-11-26

**Authors:** Pratap Kumar Patra, Rashmi Ranjan Das, Aaqib Zaffar Banday, Mini Singh, Kapil Goyal, Ankur Kumar Jindal, Surjit Singh

**Affiliations:** ^1^Department of Pediatrics, All India Institute of Medical Sciences, Patna, 801507, India; ^2^Department of Pediatrics, All India Institute of Medical Sciences, Bhubaneswar, 751019, India; ^3^Department of Pediatrics, Allergy Immunology Unit, Advanced Pediatrics Centre, Post Graduate Institute of Medical Education & Research (PGIMER), Chandigarh, 160012, India; ^4^Department of Virology, PGIMER, Chandigarh, 160012, India

**Keywords:** coronavirus, evidence-based medicine, Kawasaki disease, meta-analysis, observational study, systematic review

## Abstract

**Aim:** To study the association between non-SARS, non-MERS human coronavirus (HCoV) infections and Kawasaki disease (KD). **Methods:** Meta-analysis of observational studies published until 1 May 2021. **Results:** Out of 571 papers retrieved through database search, 10 provided data of 17,732 children. Age ranged from 2 months–14.9 years with 66% being male and 71% being complete KD. Compared with controls, there was an increased risk of developing KD in those detected to have HCoV infection (OR: 2.3 [95% CI: 1.06–4.99]; p = 0.03). The GRADE evidence for all outcomes was of ‘low-certainty’. **Conclusion:** A ‘low certainty’ of evidence suggests an increased risk of KD in children infected with HCoV. We need multi-center, prospective studies to support or refute this finding.

**PROSPERO protocol registration**: CRD42021251582.

Kawasaki disease (KD) is a common childhood vasculitis whose etiology remains unknown [1]. A complex interplay of infectious agents and environmental factors in genetically predisposed individuals is believed to give rise to this disorder [2,3]. Many viral agents that enter the body through respiratory tract have been implicated in pathogenesis of KD. This is further supported by seasonal occurrence of KD in some countries [2,3]. Peak incidence of KD in Japan and USA is observed during winter/summer and spring/winter, respectively [2,3]. Viruses that may trigger KD include adenovirus, parvovirus, influenza and parainfluenza. However, it is not known whether there is a causal relationship between these agents and KD [2,3].

Human coronaviruses (HCoV) belong to Orthocoronavirinae subfamily of Coronaviridae family [4]. Of four subfamilies, two (alpha and beta) cause respiratory and gastrointestinal infections in humans. Four human coronaviruses (HCoV-HKU1, HCoV-OC43, HCoV-229E and HCoV-NL63) cause mild respiratory tract infection and are known as non-SARS (severe acute respiratory syndrome)/non-MERS (Middle East respiratory syndrome) HCoV. On the other hand, SARS-CoV-1/2 and MERS-CoV have been associated with life-threatening infections [4].

In recent times, there has been a spate of reports on KD/KD-like disease triggered by SARS-CoV-2, termed as pediatric multisystem syndrome temporally associated with SARS-CoV-2 in EU (PIMS-TS), and multisystem inflammatory syndrome children (MIS-C) in the USA [5–7]. However, the same phenomenon has been sparingly observed in north-east Asian countries, such as Japan, Korea and Taiwan, that have much higher incidence rates of KD. This has opened up a new debate regarding the putative role of HCoV infection in evolution of KD. Systematic and narrative reviews have analyzed the association between SARS-CoV-2 and KD/KD-like illnesses [8,9]. There is, however, no published systematic review or meta-analysis that has studied the association between HCoV infection (non-SARS and non-MERS) and KD. We tried to find out if HCoV infection was increasing the risk of development of KD in children of all age groups.

## Materials & methods

This systematic review protocol is registered at PROSPERO: CRD42021251582

### Types of studies

Observational studies that aimed at studying the association of HCoV infection with KD in both community and hospital settings were included.

### Types of participants

Children of all age groups/sexes were included.

### Types of exposure

Participants in exposure group included those with HCoV infection (confirmed by either RT-PCR or serology) and KD. In control group, children had HCoV infection but no features of KD.

### Types of outcome measures

#### Primary

- Risk of development of KD in those infected with HCoVs.

#### Secondary

- Risk of development of KD in those infected with different serotypes of HCoVs (e.g., HKU1, HCoV OC43, HCoV 229E and HCoV NL63).

### Search methodology

The following databases were searched systematically from the year 1967 to 1 May 2021: PubMed/MEDLINE, Google Scholar, Embase and pre-print servers (medRxiv, bioRxiv, OSF preprints, Pre-prints.org). The PubMed/MEDLINE search strategy used the various MeSH and free text terms for ‘coronavirus’, ‘coronavirinae’, ‘novel coronavirus’, HCoV, SARS CoV-2, COVID-19, MERS-CoV, ‘Kawasaki disease’, ‘Kawasaki syndrome’, ‘mucocutaneous lymph node syndrome’, ‘observational studies’, ‘children’, ‘pediatric’, using the Boolean operators. No language restrictions were applied. The search results were reviewed to identify relevant studies.

### Data extraction

The data were extracted by using a pre-designed data extraction form and tested *a priori*. We independently extracted the following information from each study: author, year, location (country), study design, setting (hospital or community), method of recruitment, inclusion criteria, risk of bias, participants (age, sex, sample size and diagnosis), exposure (type of viruses, methods employed for diagnosis and method of ascertainment), outcomes (outcome definition, valid unit of measurement, time points of collection and reporting), lost to follow up and miscellaneous data (key conclusions, references to other studies and additional data required).

### Assessment of risk of bias in the included studies

We independently assessed quality by using the Newcastle–Ottawa scale (NOS) for observational studies [10]. It assesses the quality of studies under three major domains: selection of studies, comparability and outcome/exposure. Any disagreements were resolved by discussion.

### Dealing with missing data

The missing data were described that included dropouts in selected studies. Difference in dropout rates may lead to biased estimates of the effect size. Furthermore, bias may arise if the reasons for dropouts vary across different groups in different studies.

### Data synthesis

Data were analyzed using Review Manager (RevMan, v.5.3, The Cochrane Collaboration, Copenhagen, Denmark) [11]. Data from included studies were pooled and expressed as odds ratio (OR) with 95% CI. A p-value < 0.05 was considered statistically significant. Inter-study heterogeneity was assessed by Cochrane's Q (χ^2^ p < 0.10) and quantified by I^2^. An I^2^ ≥50% indicated ‘substantial’ heterogeneity and ≥75% indicated ‘considerable’ heterogeneity [12]. Causes of substantial or considerable heterogeneity were explored and, sensitivity and/or sub-group analyses were carried out where feasible. We used both random- and fixed-effect models to see any difference in pooled effect. We constructed the funnel plot from primary outcome to ascertain any publication bias [13].

### Grade of evidence

We used GRADE Profiler software (v.3.2) to assess certainty of evidence [14]. The software uses five parameters for rating the certainty of evidence. The parameters used are risk of bias, inconsistency of results or unexplained heterogeneity, indirectness of evidence, imprecision of results and publication bias.

## Results

### Description of studies

**Figure 1.**
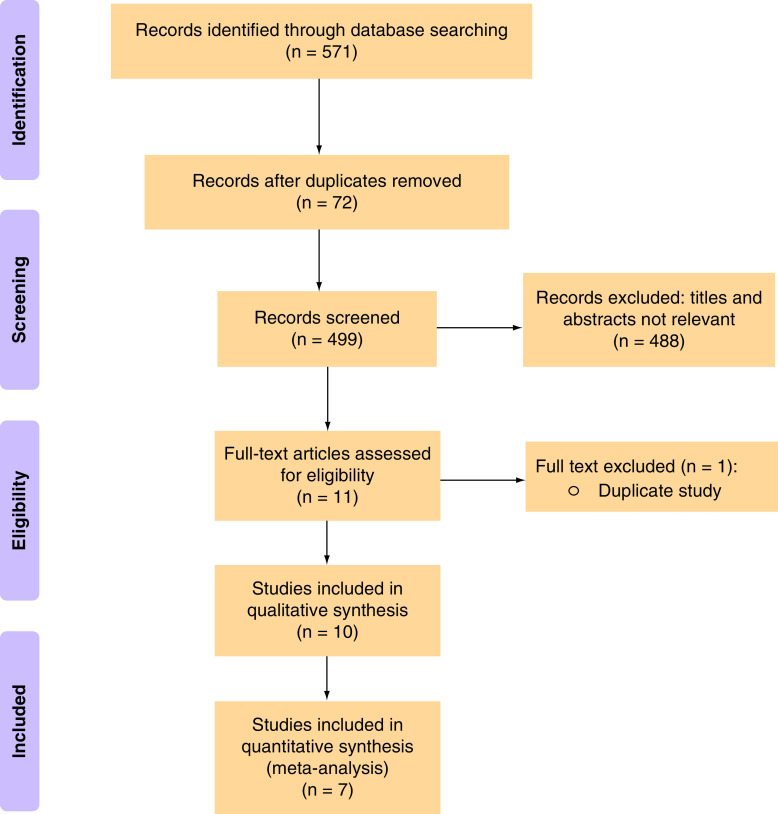
PRISMA flow diagram.

Of 571 total citations retrieved, full text of 11 papers was assessed for eligibility, and one duplicate paper was excluded (Figure 1) [15]. Of the remaining 10 eligible studies (17,732 children), we were able to do a meta-analysis of seven studies including 17,615 children (cases: 555; controls: 17,060) (Table 1). Age range of included children was 0.2–14.9 years, 66% were male and 71% had classic KD. Eight studies were case-control studies [16–23] and two were prospective studies [24,25]. The studies had been conducted in the following countries: USA (n = 5), Japan (n = 2), Taiwan (n = 2), The Netherlands (n = 1) and Germany (n = 1). Two studies employed serology (antibody assay) and immunofluorescence for diagnosis of HCoV infection, whereas the rest employed viral detection (RT-PCR and/or culture) method [20,21]. Respiratory samples (from nose, nasopharynx or throat) were used by studies that used viral detection methods. Different studies have assessed the association of different serotypes of HCoVs with KD, and details are depicted in Table 1.

**Table 1.** Characteristics of included studies.

Study (year), country	Study design, study period	Sample size (n), age and sex of children	Characteristic of cases	Characteristic of controls	Diagnostic method employed	Associated serotypes	Time from onset of symptom to obtain sample	Additional comments	Ref.
Esper *et al. *(2005), USA (single center)	Case-control study, from 2001 to 2004	n = 33 (case: 11; control: 22). Mean age (months) = case 24.4; control 23.7. Male = 9 (82%) cases	Children <5 years and report negative for RSV, influenza viruses, parainfluenza viruses and adenovirus by direct fluorescent antibody assay	For each case KD case, 2 matched controls – the first 2 children with age difference <6 months from that of the cases	RT-PCR of respiratory specimen	New haven coronavirus HCoV-NH	Median (range) days = 5 (4–13)	53 children had KD, but 11 included as their respiratory specimen was available (attrition rate = 80%). Complete KD in 10 (91%) cases	[15]
Belay *et al. *(2006), USA (single center)	Case-control study, 1999	n = 16 (case: 10; control: 6). Median age (year) = case (3.6) and control (3.3) Male = not mentioned	Cases were recruited from another study designed to look for link between KD and *Chlamydia pneumoniae*	Family members of KD patients and age-matched children attending outpatient clinics for well child visits	RT-PCR of pharyngeal specimen	HCoV-NL63	<10 days in 6 children, and in rest 4 children on days 11, 15, 16 and 37	13 children had KD, but 10 included as their specimen was available (attrition rate = 23%). Complete KD cases not mentioned	[18]
Dominguez *et al.* (2006), USA (single center)	Case-control study, from 2004 to 2005	n = 78 (case: 26; control: 52). Mean age (months) = case (46.2) and control (40.1) Male = 22 (85%) cases	Children <5 years and report negative for RSV, influenza viruses, parainfluenza viruses and adenovirus by direct fluorescent antibody assay	For each case KD case, 2 matched controls – the first 2 children with age difference <6 months from that of the cases	RT-PCR of naso-pharyngeal specimen for HCoV NL63 only	HCoV-NL63	Mean (range) days = 8.8 (4–23)	30 children had KD, but 26 included as their respiratory specimen was available (attrition rate = 13%). Complete KD in 20 (77%) cases	[21]
Lehmann *et al. *(2009), Germany (single center)	Case-control study, from 2006 to 2008	n = 54 (case: 21; control: 33). Mean age (year) = case (4.5) and control (5.2) Male = 15 (71%) cases	Children fulfilling criteria of both complete and incomplete KD were enrolled	Not described	Serological assay (IgM, IgG, IgA) by a novel line-immunoassay	HCoV-NL63	Mean (range) days = 5.5 (3–10)	No attrition. Complete KD in 12 (57%) cases	[20]
Chang *et al.* (2014), Taiwan (multi-center)	Case-control study, from 2004 to 2010	n = 452 (case: 226; control: 226). Mean age (year) = case (2.0) and control (2.0). Male = 133 (59%) cases	Children fulfilling criteria of KD were enrolled	Age- and sex-matched children who did not have preceding illness for the 2 weeks prior to enrolment	RT-PCR and viral isolation of throat and naso-pharyngeal specimen	HCoV-NL63 HCoV-229E HCoV-OC43	Mean (SD) days = 7.5 (2.4)	No attrition. Complete KD cases not mentioned	[22]
Shirato*et al. *(2014), Japan (single center)	Case-control study, from 2001 to 2002	n = 134 (case: 30; control: 104). Mean age (year) = case (2.3) and control (2.3). Male = 20 (67%) cases	Children fulfilling criteria of KD were enrolled	Age- and sex-matched healthy children	Serological assay (immunofluorescence and viral neutralization)	HCoV-NL63 HCoV-229E	Mean (range) days = 4.5 (1–7)	No attrition. Complete KD cases not mentioned	[19]
Turnier *et al.* (2015), USA (single center)	Case-control study, from 2009 to 2013	n = 16,607 (case: 192; control: 16,415). Mean age (years) = case (2.9) and control (not mentioned) Male = 111 (58%) cases	Children fulfilling criteria of KD were enrolled	Those with available naso-pharyngeal specimen during the study period	RT-PCR of naso-pharyngeal specimen	HCoV-NL63 HCoV-229E HCoV-OC43 HCoV-229E	Median (range) days = 6 (5–8)	222 children had KD, but 192 included as their respiratory specimen was available (attrition rate = 14%). Complete KD in 132 (78%) cases	[17]
Shimizu *et al.* (2005), USA and The Netherlands (multi-center)	Prospective study, from 2000 to 2005	n = 48. Age = 2 months to 10 years. Male = 42 (88%)	Children fulfilling criteria of KD were enrolled	N/A	RT-PCR of throat and naso-pharyngeal specimen	HCoV-NL63	Median (range) days = 7 (3–15)	Only 1 patient was positive for HCoV. No attrition. Complete KD cases not mentioned	[24]
Chang *et al.* (2006), Taiwan (multi-center)	Prospective study, from 2004 to 2005	n = 53. Age (mean) = 2.3 years. Male = 27 (51%)	Children fulfilling criteria of complete and incomplete KD were enrolled	N/A	RT-PCR of throat, rectal and naso-pharyngeal specimen	HCoV-NH HCoV-NL63	M (S) days = 8.2 (2.8)	None of the patients was positive for HCoV. No attrition. Complete KD in 52 (98%) cases	[23]
Ebihara *et al.* (2005), Japan	Case-control study, from 2002 to May 2003	n = 19 Mean Age = 22.6 months 4 months to 5 years	Children with KD were included	208 children with respiratory tract infection	RT-PCR of nasopharyngeal sample	Hcov-NH	Within 7 days	None of the patient with KD had HCoV. No mention about complete or incomplete KD	[16]

KD: Kawasaki disease; N/A: Not applicable; RT-PCR: Real-time polymerase chain reaction; RSV: Respiratory syncytial virus; SD: Standard deviation.

### Risk of bias in included studies

None of the studies was having a risk of bias in selection of cases, but four were at a high risk of bias for selection of controls [17,18,20,21]. Two studies were at high risk of bias for comparability of cases and controls [18,21]. All studies were having a low risk of bias for exposure parameters (Table 2).

**Table 2.** Risk of bias (RoB) table.

Study (year)	Selection				Comparability	Exposure			Total
	Is the case definition adequate?	Representativeness of the cases	Selection of controls	Definition of controls	Comparability of cases and controls on the basis of the design or analysis	Ascertainment of exposure	Same method of ascertainment for cases and controls	Nonresponse rate	
Belay *et al.* (2005)	***_				*	**_			******
Chang *et al.* (2014)	***_				*	***			*******
Dominguez *et al.* (2006)	***_				*	**_			******
Ebihara *et al.* (2005)	*_*_				*	**_			*****
Esper *et al.* (2005)	***_				*	**_			******
Shirato *et al.* (2014)	**__				*	**_			*****
Turnier *et al.* (2015)	**__				_	**_			****
Lehmann *et al.* (2009)	**__				_	_**			****

This form has been designed to assess case control studies based on: (1) Selection of subjects (maximum of 4 stars were given one against each item); (2) Selection of controls (maximum of 2 stars were given) and (3) Assessment of exposure (maximum of 3 stars were given one against each numbered item).

### Effect of exposures

#### Primary outcome

##### Risk of development of KD in those infected with novel HCoV

**Figure 2.**
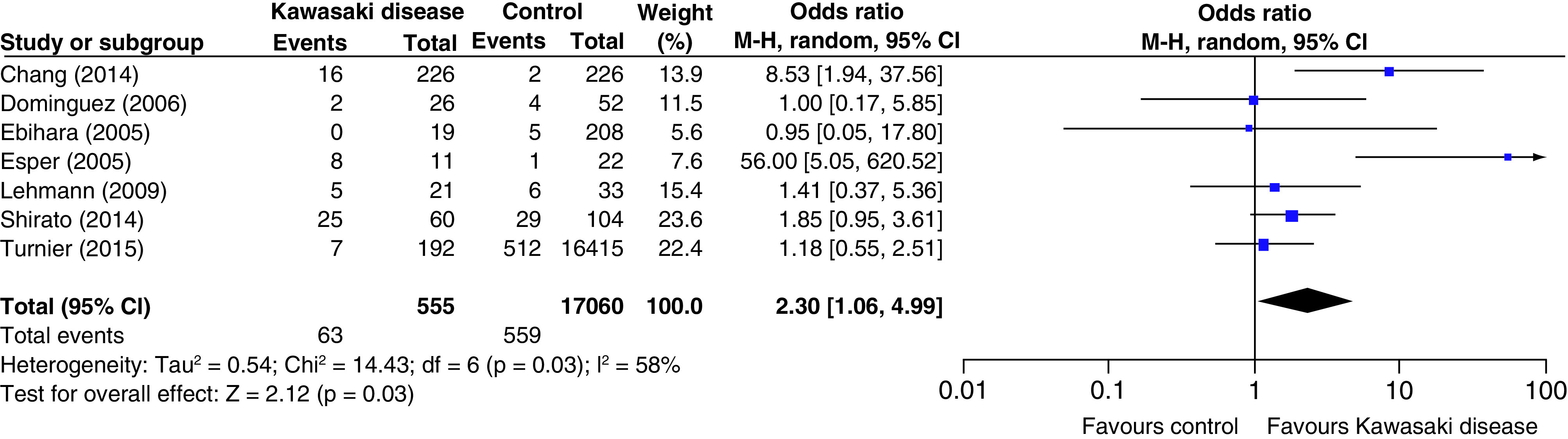
Primary outcome (odds of developing Kawasaki disease after infection with human coronavirus.

Overall analysis: Seven studies were able to contribute data to the pooled analysis. Both fixed- and random-effect models were employed to see whether the findings were significant or not, as significant heterogeneity was noted among included studies (I^2^ = 58%; p = 0.03). There was an increased risk (odds) of developing KD in those detected to have infection with HCoV as found in both the models (random-effect model: OR: 2.3 [95% CI: 1.06–4.99]; p = 0.03 [Figure 2]; and fixed-effect model: OR: 2.16 [95% CI: 1.47–3.15]; p = <0.0001).

Sensitivity analysis: We did sensitivity analysis as one small study was contributing to the heterogeneity [16]. The pooled data showed decreased heterogeneity (21%) with an increased risk (odds) of developing KD in those detected to have infection with HCoV [OR: 1.86 (95% CI: 1.24 to 2.77); p = <0.002].

Subgroup analysis: we separately analyzed studies employing different diagnostic methods for HCoV (RT-PCR/viral detection or antibody assay). Overall analysis was not significant for studies employing either RT-PCR/viral detection (OR: 3.22 [95% CI: 0.79 to 13.08]; p = 0.1) or antibody assay (OR: 1.75 [95% CI: 0.96 to 3.18]; p = 0.07). As there was significant heterogeneity among studies, we did sensitivity analysis by removing one study [16]. Pooled data was significant without any remarkable heterogeneity (OR 1.96 [95% CI: 1.15 to 3.33]; p = 0.01).

#### Secondary outcome

##### Risk of development of KD in those infected with different serotypes of HCoV

We separately analyzed data from included studies that reported different serotypes of HCoV (NL63, OC43, 229E). However, none of the serotypes was individually associated with risk of development of KD.

###### Publication Bias

**Figure 3.**
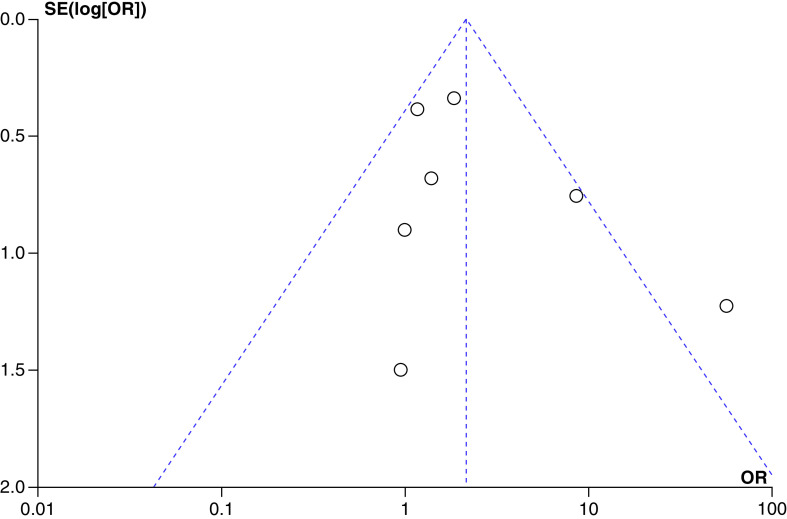
Funnel plot assessing publication bias in the included studies. OR: Odds ratio.

Funnel plot was asymmetrical showing publication bias (Figure 3).

###### Grade (certainty) of evidence

Evidence generated was of ‘low certainty’ for all outcomes (primary and secondary). Likely reasons were heterogeneity among studies, poor methodological design and selective outcome reporting. A detailed analysis of the summary of evidence for primary outcome is provided in Table 3.

**Table 3.** GRADE table. Association of novel human coronavirus compared with no human coronavirus in children with Kawasaki disease.

Patient or population: children with Kawasaki disease Settings: community and Hospital Exposure: HCoV Comparison: No HCoV					
Outcomes	Illustrative comparative risks^†^ (95% CI)		Relative effect (95% CI)	Number of participants (studies)	Certainty of the evidence (GRADE)
	Assumed risk	Corresponding risk			
	No coronavirus	Coronavirus			
Association of HCoV with KD (all study data)	Study population		OR: 2.3 (1.06–4.99)	17,615 (7 studies)	⊕⊝⊝⊝ Low^‡^,^§^,^¶^
	32 per 1000	81 per 1000 (34–179)			
Association of HCoV with KD (sensitive analysis)	Study population		OR: 1.86 (1.24–277)	17,582 (6 studies)	⊕⊕⊝⊝ Low^‡^,^§^,^¶^
	32 per 1000	60 per 1000 (41–89)			

GRADE Working Group grades of evidence.

High quality: Further research is very unlikely to change our confidence in the estimate of effect.

Moderate quality: Further research is likely to have an important impact on our confidence in the estimate of effect and may change the estimate.

Low quality: Further research is very likely to have an important impact on our confidence in the estimate of effect and is likely to change the estimate.

Very low quality: We are very uncertain about the estimate.

† The basis for the assumed risk (e.g., the median control group risk across studies) is provided in footnotes. The corresponding risk (and its 95% CI) is based on the assumed risk in the comparison group and the relative effect of the intervention (and its 95% CI).

‡ Case-control design of included studies.

§ Significant statistical heterogeneity.

¶ Asymmetry in funnel plot noted.

KD: Kawasaki disease; OR: Odds ratio.

## Discussion

### Summary of evidence

After an extensive search of the literature, we included ten studies with data of 17,732 children. As compared with controls, those having documented infection with HCoV were at a higher risk of developing KD. No particular serotype of HCoV was found to be associated with this increased risk. The evidence, however, is of ‘low certainty’.

In a case-control study published in 2005, Esper *et al.* first demonstrated the association between HCoV infection and KD [16]. Chang *et al.* confirmed this association in 2014 [23]. However, further case-control studies failed to replicate this association [17,20,21] which was likely due to relatively small sample sizes in the two older studies. Of the ten studies included in the present review, eight used RT-PCR for detection of HCoV, and two used serological assays. Use of serological assays may have been the reason for low detection rates of HCoV in these studies, as sensitivity of serological assays is lower than RT-PCR. Furthermore, there is a significant variability among studies based upon the interval between diagnosis of KD and RT-PCR testing for HCoV infection. Additionally, only a few studies have documented presence of concomitant respiratory symptoms (due to HCoV infection) and occurrence of KD.

In the present meta-analysis, we have analyzed the association of HCoV infections with KD. This has not been evaluated comprehensively to date. Recognition of association between HCoV with KD predates the KD or KD-like illnesses (in the form of multisystem inflammatory syndrome in children) seen in the ongoing SARS-CoV-2 pandemic by about a decade and a half. KD, KD-like illness (MIS-C/PIMS-TS) and severe COVID-19 infection induced vasculitis represent a similar disease spectrum characterized by a dysregulated immune response. It suggests that SARS-CoV-2 may just represent a ‘new kid on the block’ of various coronavirus infections associated with KD. In fact, KD has also been reported in association with the H1N1-pdm09 influenza infection that resulted in a pandemic a decade ago [26]. Besides, several other infectious illnesses have also been associated with KD [2]. Hence, there is a distinct possibility that there may be a surge in cases of KD or KD-like illnesses in future pandemics as well.

Exact etiology of KD remains an enigma. Many infectious illnesses can trigger KD in a genetically susceptible host. Of the various microbial agents, respiratory viruses have come up as the most important triggers for KD [2]. Causality has not been proven for any of the associated infectious agents to date, although, a KD-specific RNA respiratory virus has been postulated to exist based largely on autopsy studies [27]. Epidemiological profile of KD also favors the theory of an infectious trigger as it is predominantly seen in children below 5 years, is associated with seasonal clustering of cases with occasional epidemics, and the clinical features (such as fever, rash, cervical lymphadenopathy) mimic an infectious disorder.

It is plausible that HCoV infections may trigger KD illness in susceptible individuals. A number of genetic polymorphisms have been associated with an increased risk of developing KD in certain populations. Variability in HCoV-associated KD in different studies can therefore be easily explained by genetic differences among study populations [28]. It is possible that HCoV may be a risk factor for KD in populations with certain polymorphism(s) and not in others.

The exact pathogenesis of KD following HCoV infection is poorly understood and multifactorial. Endothelial dysfunction or inflammation is the hallmark of both KD and KD-like conditions that follow HCoV and SARS-CoV2 infection. HCoV has spike like protein that binds to angiotensin converting enzyme 2 (ACE2) receptors on surface of the target cell. A systematic review and meta-analysis have documented a significant risk of KD with ACE2 polymorphism [29]. Additionally, ACE2 polymorphisms have also been associated with other systemic vasculitides. SARS-CoV-2 has been shown to modulate ACE2 expression resulting with elevated TNF-α production [30]. Thus, modulation of ACE2 expression as a result of coronavirus infection or genetic polymorphisms may be one of the unifying pathogenetic mechanisms between the KD/KD-like illnesses following coronavirus infection and KD not associated with coronavirus infection.

### Strengths & limitations

A comprehensive literature search, robust analysis and grading of evidence are strengths of the present review. Inclusion of only retrospective studies into the pooled analysis is a limitation (albeit unavoidable) of our review as the small sample size and retrospective nature may have introduced bias to overall estimate of results. Another limitation is that only three authors were involved in search methodology, risk of bias assessment and data extraction. This overlapping role might have introduced some bias in the review process. Furthermore, all studies were hospital based and were conducted mostly in developed countries. Hence, it may not be prudent to extrapolate the findings of this meta-analysis to the community at large.

## Conclusion

A ‘low certainty’ of evidence suggests an increased risk of KD in children infected with HCoV. We need multi-center, prospective studies to support or refute this finding. The findings will be of great public health importance as KD/KD-like illnesses are closely associated with SARS-CoV-2 pandemic.

## Future perspective

There should be elucidation of actual link between Kawasaki disease, and human coronaviruses (both SARS and non-SARS) at molecular level so that targeted therapy can develop as a form of prophylaxis.

**Summary points**

- Many studies have analyzed the association between SARS-CoV-2 and Kawasaki disease (KD)/KD-like illnesses with conflicting results, however, systematic review(s) or meta-analyses that have studied the association between human coronavirus (HCoV) infection (non-SARS and non-MERS) and KD are lacking.
- We included data of 17,732 children from ten observational studies, there was an increased risk (odds) of developing KD in those detected to have infection with HCoV (random-effect model: OR [odds ratio]: 2.3 [95% CI: 1.06–4.99]; fixed-effect model: OR: 2.16 [95% CI: 1.47–3.15]).
- None of the HCoV serotype was individually associated with risk of development of KD.
- ‘Low certainty’ evidence suggests an increased risk of KD in children infected with HCoV. Likely reasons were heterogeneity among studies, poor methodological design and selective outcome reporting.
- Publication bias was noted among the published studies.
- We need multi-center, prospective studies to support or refute this finding. The findings will be of great public health importance as KD/KD-like illnesses are closely associated with SARS-CoV-2 pandemic.
